# Characteristics of elderly patients with polypharmacy who refuse to participate in an in-hospital deprescribing intervention: a retrospective cross-sectional study

**DOI:** 10.1186/s12877-018-0788-1

**Published:** 2018-04-17

**Authors:** Junpei Komagamine, Kenichi Sugawara, Kazuhiko Hagane

**Affiliations:** 1 Department of Internal Medicine, National Hospital Organization Tochigi Medical Center, 1-10-37, Nakatomatsuri, Utsunomiya, Tochigi 3208580 Japan; 2 Department of Pharmacy, National Hospital Organization Tochigi Medical Center, 1-10-37, Nakatomatsuri, Utsunomiya, Tochigi 3208580 Japan; 3 Department of Pediatric Surgery, National Hospital Organization Tochigi Medical Center, 1-10-37, Nakatomatsuri, Utsunomiya, Tochigi 3208580 Japan

**Keywords:** Deprescribing, Polypharmacy, Potentially inappropriate medications

## Abstract

**Background:**

Few studies have evaluated the characteristics of elderly patients with polypharmacy refusing deprescribing. The aim of this study was to evaluate the prevalence of potentially inappropriate medication (PIM) use in elderly patients accepting and refusing a deprescribing intervention and to investigate factors associated with deprescribing refusal.

**Methods:**

We conducted a retrospective cross-sectional study by analyzing the electronic medical records from a single hospital. All consecutive patients aged 65 years or older who reported the use of five or more medications upon admission to the orthopedic ward from January 2015 to December 2016 and who were approached by a pharmacist for polypharmacy screening were included. Patients who had provided consent for the deprescribing intervention by the internal medicine physicians were defined as the acceptance group, and patients who did not were defined as the refusal group. The primary outcome was the use of any PIMs at admission, based on the 2015 American Geriatric Society Beers Criteria. Using multivariable logistic regression, predictive factors of refusing deprescribing were also evaluated.

**Results:**

During the study period, 136 patients were eligible. Of those, 82 patients (60.3%) accepted the deprescribing intervention, and 54 patients (39.7%) declined the intervention. The mean age of all the patients was 81.1 years, and the mean number of medications at admission was 9.3. The overall proportion of patients taking any PIMs at admission was 77.2%. The proportion of patients taking any PIMs at admission was not different between the acceptance and refusal groups (78.0% and 75.9%, respectively; *p* = 0.84). None of the measured characteristics, including age, gender, residential status, comorbidity, alcohol use, smoking status, number of medications, or number of PIMs, were found to be associated with deprescribing refusal.

**Conclusion:**

The prevalence of any PIM use did not differ among elderly orthopedic patients with polypharmacy according to refusal or acceptance of the deprescribing intervention. Furthermore, none of the analyzed characteristics were found to be associated with deprescribing refusal. Given the high prevalence of PIM use, a strategy is needed for combating polypharmacy among elderly patients reluctant to undergo deprescribing.

## Background

Polypharmacy, a common problem among elderly patients due to multi-morbidities [1, 2], is associated with inappropriate prescribing [3], which can be harmful [4]. Recently, deprescribing has been suggested to reduce inappropriate polypharmacy among elderly patients [5–7]. Deprescribing is a systematic process of identifying and discontinuing drugs in instances in which existing or potential harms outweigh existing or potential benefits within the context of an individual patient’s care goals, current level of functioning, life expectancy, values, and preferences [7]. Although it is uncertain whether deprescribing in elderly patients improves clinical outcomes, this strategy can safely and effectively reduce the use of potentially inappropriate medications (PIMs) [8–10].

Nonetheless, there are some barriers for deprescribing in elderly patients with inappropriate polypharmacy. These barriers result from patient-, prescriber- and system-related factors [11–13]. Patient factors include fear of consequences of cessation and feeling that a medication is currently beneficial for a condition. Past randomized controlled studies for deprescribing in elderly patients with polypharmacy have reported that over 30% of eligible patients refused to undergo deprescribing and were thus excluded from the study [14–16]. This refusal rate seems overly high given that other recent studies evaluating elderly peoples’ attitudes regarding polypharmacy reported that 80–92% of participants are willing to have one or more medications deprescribed if their doctor said it was possible [17–19]. This gap might reflect the difference between real-world practice and survey studies. Even patients who indicate they prefer deprescribing in a survey might refuse when actually recommended to stop their medications. However, these studies lacked information regarding the appropriateness of medications among patients who refused deprescribing; this lack of information is problematic if the PIM prevalence for polypharmacy patients who refuse deprescribing is similar to or higher than the PIM prevalence for those who accept deprescribing. To our knowledge, no studies have evaluated the difference in PIM prevalence in elderly patients with polypharmacy who refuse or accept deprescribing. Furthermore, factors associated with deprescribing refusal in elderly patients with polypharmacy have not been evaluated in a real-world practice. Thus, our aim was to evaluate PIM prevalence in elderly patients with polypharmacy who accept or refuse deprescribing as well as factors associated with deprescribing refusal.

## Methods

### Study design and participants

A retrospective cross-sectional study was conducted using the database of the National Hospital Organization Tochigi Medical Center, with data collected from January 1, 2015 to December 31, 2016. The National Hospital Organization Tochigi Medical Center is a 350-bed acute care hospital in the Tochigi prefecture of Japan. All consecutive patients aged 65 years or more with 5 or more medications at admission who were admitted to the orthopedic ward in our hospital and who were approached by a pharmacist for polypharmacy screening were included. Patients admitted to other wards or aged less than 65 years were excluded. We also excluded patients with a second admission to the orthopedic ward during the study period. The requirement for individual informed consent was formally waived by the Medical Ethical Committee of National Hospital Organization Tochigi Medical Center because the data were collected from medical records and the patients were not contacted. The study protocol was approved by the institutional research ethics committee of National Hospital Organization Tochigi Medical Center (No. 28-20) and was carried out in accordance with the Declaration of Helsinki.

Beginning in January 2015, our hospital started screening and intervening to reduce inappropriate or unnecessary medication use for elderly patients hospitalized in the orthopedic ward. Pharmacists attempted to contact elderly patients prescribed five or more medications at admission and their families to inform them of the intervention to reduce inappropriate or unnecessary medication use. The patients the pharmacists could contact and who provided written consent to receive the polypharmacy intervention were scheduled for an appointment with an internal medicine physician as soon as possible. The physicians took a history from the patients, performed a physical and neurological examination, evaluated the appropriateness of the polypharmacy, changed medications as appropriate and followed up with patients after the consultation until they were discharged. Thus, patients who had given written consent to receive the intervention were defined as the acceptance group, whereas those who did not were defined as the refusal group. This deprescribing intervention was part of the usual care regimen in our hospital rather than a separate research study. However, we did obtain written consent from patients to conduct the intervention as part of the usual care regimen because this practice is unfamiliar in Japan.

### Measurements

#### Characteristics

Information on age, gender, primary diagnosis for admission, social history, past medical history, Charlson Comorbidity Index (CCI) [20], and medications was retrieved from medical records taken at the time of each patient’s first admission. Medications were determined based on a comprehensive medication history performed by a pharmacist. Medications included oral medications, inhalers, and injections. As-needed medications were also included, but eye drops, intranasal infusers, over-the-counter (OTC) drugs, and topical medications were excluded because the deprescribing intervention of our hospital did not target these medications.

#### Outcome measures

The primary outcome was the use of any PIMs at admission. PIMs were defined based on the 2015 Beers Criteria of the American Geriatric Society (AGS) [21]. Of the five parts of the Beers Criteria, we used only two parts: PIM use in older adults and PIM use in older adults due to drug-disease or drug-syndrome interactions that may exacerbate the disease or syndrome. The secondary outcome was the number of total medications and PIMs at admission.

### Statistical analysis

We estimated that a sample of 132 patients would provide the study with a power of at least 80% to show an absolute difference of 25% for the primary outcome between the acceptance and refusal groups, assuming that approximately half of eligible patients would decline the intervention (based on previous studies [14–16]) and that the proportion of patients taking any PIMs was 50% (based on previous studies [22, 23]) in the acceptance group.

The baseline characteristics of the study population were compared using Fisher’s exact test for categorical variables and Student’s t-test for continuous variables. The proportion of patients taking any PIMs at admission among the refusal group was compared with those among the acceptance group using Fisher’s exact test. To identify the determinants of refusal for deprescribing, we also conducted multivariable analysis using binary logistic regression to examine the association between select variables and refusal. The following variables were entered in the logistic regression model: age, gender, CCI, residential status, dementia, depression, insomnia, current smoker, regular drinker, number of medications at admission, and number of PIMs at admission. These analyses were carried out using Excel statistical software package version 2.11 (Bellcurve for Excel; Social Survey Research Information Co., Ltd., Tokyo, Japan), and the level of significance was set at 5%.

## Results

We screened 178 orthopedic patients at least 65 years old with 5 or more medications during the study period; 40 were excluded because the pharmacists could not contact them to discuss the polypharmacy intervention, and 2 were excluded because of a second admission during the study period. Thus, a total of 136 patients were included in the final analysis. Among these patients, 54 (39.7%) refused the deprescribing intervention, and 82 patients (60.3%) provided consent for the deprescribing intervention.

The baseline characteristics of the patients in each group are presented in Table 1. For all 136 patients, the mean age was 81.1 years, 34 (25.0%) were men, the mean CCI was 1.9, 26 (19.1%) had dementia, 17 (12.5%) were institutional residents, 10 (7.4%) were current smokers, and 15 (11.0%) were regular drinkers. The mean number of medications at admission was 9.3. These demographic features were similar between the acceptance and refusal groups; however, the refusal group tended to have a higher proportion of current smokers compared with the acceptance group, although this association was not statistically significant (*p* = 0.051).

**Table 1:** Characteristics of the 136 included orthopedic patients

	Deprescribing for polypharmacy		*P*-value^a^
	Acceptance	Refusal	
	*N* = 82	*N* = 54	
Age, mean ± SD	80.3 ± 7.5	82.3 ± 7.4	0.14
Men, *n* (%)	22 (26.8)	12 (22.2)	0.69
Institutional resident, *n* (%)	10 (12.2)	7 (13.0)	1.00
CCI, mean ± SD	1.9 ± 1.5	1.7 ± 1.7	0.50
Current smoker, *n* (%)	3 (3.7)	7 (13.0)	0.05
Regular drinker, *n* (%)	7 (8.5)	8 (14.8)	0.28
Number of medications at admission, mean ± SD	9.6 ± 3.2	9.0 ± 2.3	0.22
Primary reason for admission, *n* (%)			
Fracture	56 (68.3)	41 (75.9)	0.44
Osteoarthritis	6 (7.3)	7 (13.1)	0.37
Spinal stenosis	9 (11.0)	3 (5.6)	0.36
Other	11 (13.4)	3 (5.6)	0.16
Past medical history, *n* (%)			
Depression	6 (7.3)	8 (14.8)	0.25
Insomnia	28 (34.1)	21 (38.9)	0.59
Dementia	15 (18.3)	11 (20.4)	0.83
Stroke	23 (28.0)	10 (18.5)	0.23
Myocardial infarction	8 (9.8)	6 (11.1)	0.78
Heart failure	8 (9.8)	3 (5.6)	0.53
Diabetes mellitus	19 (23.2)	11 (20.4)	0.83
Asthma or COPD	10 (12.2)	4 (7.4)	0.57
Rheumatological disease	10 (12.2)	6 (11.1)	1.00
Active cancer	7 (8.5)	2 (3.7)	0.20

^a^Fisher’s exact tests or Student’s t-tests were used for comparisons between the acceptance and refusal groups. The threshold for statistical significance was set at *p* < 0.05

Table 2 shows the total number of PIMs and the proportions of patients taking any PIMs at admission in each group according to drug subcategory. For all patients, the mean number of PIMs at admission was 1.5, and the proportion of patients taking any PIMs at admission was 77.2%. The most common categories of PIMs were benzodiazepines (*N* = 48, 35.3%), followed by proton pump inhibitors (*N* = 45, 33.1%), non-steroidal anti-inflammatory drugs (*N* = 14, 10.3%) and hypnotics (*N* = 13, 9.6%). There were no significant differences in the proportion of patients taking any PIMs at admission between the refusal group and the acceptance group (75.9% and 78.0%, respectively; *p* = 0.84). This proportion did not differ between the two groups for each PIM category at admission.

**Table 2:** Prevalence of PIM^a^ use according to drug subcategory at admission among the 136 included orthopedic patients

	Deprescribing for polypharmacy		*P*-value^b^
	Acceptance	Refusal	
	*N* = 82	*N* = 54	
Number of PIMs, mean ± SD	1.5 ± 1.2	1.6 ± 1.3	0.52
Any PIMs, *n* (%)	64 (78.0)	41 (75.9)	0.84
Category of PIMs, *n* (%)			
Benzodiazepines	30 (36.6)	18 (33.3)	0.72
Proton pump inhibitors	26 (31.7)	19 (35.2)	0.71
NSAIDs	7 (8.5)	7 (13.0)	0.41
Hypnotics	8 (9.8)	5 (9.3)	1.00
Anticholinergics	3 (3.7)	6 (11.1)	0.16
Anticonvulsive drugs	4 (4.9)	5 (9.3)	0.48
Antipsychotics	7 (8.5)	1 (1.9)	0.15
Opioids	2 (2.4)	5 (9.3)	0.11
Antidepressants	3 (3.7)	4 (7.4)	0.43
Ticlopidine or dipyridamole	5 (6.1)	1 (1.9)	0.40
Peripheral alpha-1 blocker	2 (2.4)	4 (7.4)	0.22

^a^PIMs were defined based on the 2015 American Geriatric Society Beers Criteria

^b^Fisher’s exact tests or Student’s t-tests were used for comparisons between the acceptance and refusal groups. The threshold for statistical significance was set at *p* < 0.05

Table 3 shows the results of binary logistic regression analysis to predict refusal of the deprescribing intervention. In both the univariable and multivariable analyses, none of the 11 variables examined were associated with the refusal of the deprescribing intervention.

**Table 3:** Summary of logistic regression results to predict deprescribing refusal

	Odds ratio (95% confidence interval)	
	Univariable	Multivariable^a^
Increasing age^b^	1.04 (0.99-1.09)	1.05 (0.99-1.10)
Male gender	0.78 (0.35-1.75)	0.78 (0.32-1.89)
Institutional resident	1.07 (0.38-3.01)	0.58 (0.16-2.12)
Increasing CCI^b^	0.93 (0.74-1.16)	1.00 (0.77-1.31)
Dementia	1.14 (0.48-2.72)	0.91 (0.29-2.85)
Depression	2.20 (0.72-6.75)	2.05 (0.58-7.23)
Insomnia	1.23 (0.60-2.50)	1.01 (0.42-2.48)
Regular drinker	1.86 (0.63-5.48)	1.80 (0.55-5.89)
Current smoker	3.92 (0.97-15.91)	3.76 (0.86-16.45)
Increasing number of medications at admission^b^	0.92 (0.81-1.05)	0.90 (0.78-1.05)
Increasing number of PIMs^c^ at admission^b^	1.09 (0.83-1.44)	1.13 (0.75-1.71)

^a^The following variables were adjusted: age, gender, CCI, residential status, dementia, depression, insomnia, current smoker, regular drinker, number of medications at admission, and number of PIMs at admission

^b^Continuous variable used

^c^PIMs were defined based on the 2015 American Geriatric Society Beers Criteria

## Discussion

The results of this study showed that the proportion of patients taking any PIMs at admission did not differ between elderly patients refusing and accepting the deprescribing intervention. Furthermore, the number of PIMs at admission was not associated with a reduced risk of refusal for the intervention.

To our knowledge, this is the first study to evaluate the differences in the proportion of patients taking any PIMs between elderly patients with polypharmacy who accept or refuse a deprescribing intervention. Our findings indicate that elderly patients refusing deprescribing intervention are subjected to inappropriate polypharmacy as frequently as those accepting it. Furthermore, the refusal rate for the deprescribing intervention was 39.7%, which is consistent with past studies showing that more than 30% of patients screened for an intervention for deprescribing had declined to participate [14–16]. Given the high prevalence of polypharmacy among elderly patients, this high rate of refusal for deprescribing is disappointing. To resolve the problem of inappropriate polypharmacy among elderly patients, in addition to providing intervention for those willing to receive it, a strategy is needed for inappropriate polypharmacy among elderly patients reluctant to receive deprescribing.

Multivariable analysis was used to examine variables predicting refusal of the deprescribing intervention, but no variables were identified. Considering that factors, such as PIM use, age and CCI, could not predict willingness to deprescribe, it is possible that other uncaptured factors, such as the values and preferences of patients, are involved. Therefore, further studies are warranted to explore the association between these factors and deprescribing refusal among elderly patients with polypharmacy. In this study, the proportion of patients who took NSAIDs or opioids tended to be higher in the refusal group than in the acceptance group. Although these differences were not statistically significant, this study may be underpowered due to its small sample size. Given that the success rate of deprescribing intervention varies according to drug category in a past study [16], patients’ concern that the specific medication they prefer to continue could be targeted for deprescribing may also affect their decision on the deprescribing intervention. Thus, the effect of specific medications also needs to be investigated in the future.

### Limitations

Our results should be interpreted in the context of several limitations. First, this study utilized a retrospective design. Furthermore, this study was limited to a single center and to elderly patients admitted to the orthopedic ward who were taking five or more medications; as such, the results may not be easily generalized. Therefore, these findings should be confirmed by future prospective studies in other settings. Second, we excluded OTC drugs. Therefore, the prevalence of PIM use may be underestimated. Third, our assessment did not include potential prescription omissions [24]. Fourth, our deprescribing intervention did not target several medications, such as eye drops and topical medications. Therefore, our findings cannot be generalized to the medications excluded from this study. Fifth, the small sample size resulted in wide 95% confidence intervals for odds ratios for several variables such as smoking status, alcohol use, and depression. Therefore, the association between these variables and deprescribing refusal must be investigated in future studies with a larger sample size. Sixth, the pharmacists could not screen all consecutive orthopedic patients with polypharmacy that were seen over the study period. Therefore, the results might be affected by selection bias. Seventh, refusal to participate in a deprescribing intervention when at a hospital may differ from refusing deprescribing by a primary care physician when the patient is medically stable. Furthermore, patients who accepted the intervention may not have been willing to discontinue all their PIMs. Eighth, given that 19.1% of patients had dementia, caregiver-related factors might have affected the results. However, it should be noted that dementia was not a predictive factor for intervention refusal in the multivariable analysis. Ninth, we did not evaluate several important factors, such as the patients’ knowledge of PIM or the values and preferences of the patients, which could affect their decision regarding whether to participate in the intervention. Furthermore, the selected variables in the multivariable analysis were not based on the conceptual model [11]. Finally, a tone of “research” introduced during the informed consent procedure for the deprescribing intervention may affect the rate of refusal, although the deprescribing intervention was a component of the usual care regimen in our hospital.

## Conclusion

The proportion of patients taking any PIMs was not different among elderly orthopedic patients taking five or more medications who refused or accepted the deprescribing intervention. Furthermore, none of the characteristics measured were found to be associated with willingness to participate in the deprescribing intervention. Given the high prevalence of PIM use, a strategy is needed for elderly patients with polypharmacy who are reluctant to undergo deprescribing.

## Abbreviations

CCI: Charlson comorbidity index
COPD: Chronic obstructive pulmonary disease
NSAIDs: Non-steroidal anti-inflammatory drugs
PIM: Potentially inappropriate medication
SD: Standard deviation
